# Immunochemical faecal occult blood test: number of samples and positivity cutoff. What is the best strategy for colorectal cancer screening?

**DOI:** 10.1038/sj.bjc.6604864

**Published:** 2009-01-13

**Authors:** G Grazzini, C B Visioli, M Zorzi, S Ciatto, F Banovich, A G Bonanomi, A Bortoli, G Castiglione, L Cazzola, M Confortini, P Mantellini, T Rubeca, M Zappa

**Affiliations:** 1 Department of Screening, ISPO Cancer Prevention and Research Institute Florence, Viale Volta 171, 50131 Florence, Italy; 2 Venetian Tumour Registry, Istituto Oncologico Veneto Via Gattamelata 64, 35128 Padova, Italy; 3 Local Health Unit no. 4, Veneto Region, Via Rasa 9, 36016 Thiene, Italy; 4 Local Health Unit no. 22, Veneto Region, Via Ospedale 16, 37012 Bussolengo, Italy; 5 Local Health Unit no. 2, Veneto Region, Via Borgo Ruga 30, 32032 Feltre, Italy

**Keywords:** immunochemical test, colorectal neoplasms, faecal occult blood test, screening, cutoff

## Abstract

Immunochemical faecal occult blood tests have shown a greater sensitivity than guaiac test in colorectal cancer screening, but optimal number of samples and cutoff have still to be defined. The aim of this multicentric study was to evaluate the performance of immunochemical-based screening strategies according to different positivity thresholds (80, 100, 120 ng ml^−1^) and single *vs* double sampling (one, at least one, or both positive samples) using 1-day sample with cutoff at 100 ng ml^−1^ as the reference strategy. A total of 20 596 subjects aged 50–69 years were enrolled from Italian population-based screening programmes. Positivity rate was 4.5% for reference strategy and 8.0 and 2.0% for the most sensitive and the most specific strategy, respectively. Cancer detection rate of reference strategy was 2.8‰, and ranged between 2.1 and 3.4‰ in other strategies; reference strategy detected 15.6‰ advanced adenomas (range=10.0–22.5‰). The number needed to scope to find a cancer or an advanced adenoma was lower than 2 (1.5–1.7) for the most specific strategies, whereas it was 2.4–2.7, according to different thresholds, for the most sensitive ones. Different strategies seem to have a greater impact on adenomas rather than on cancer detection rate. The study provides information when deciding screening protocols and to adapt them to local resources.

Faecal occult blood testing (FOBT)-based screening has been proven effective in reducing mortality from CRC (Hewitson *et al*, 2007). Recently, immunochemical tests (IFOBT) have been shown to be more sensitive than classic guaiac testing, and, as IFOBTs are specific for human haemoglobin (Hb), they do not require dietary restrictions, thus potentially improving screening acceptability. Moreover, some studies (Castiglione *et al*, 2000; van Rossum *et al*, 2008) suggested that 1-day IFOBT is more accurate (in terms of sensitivity/specificity ratio) compared to 3-day guaiac testing. Single sampling would enhance screening feasibility, and likely compliance, which has always been a critical factor (Vernon, 1997; van Rossum *et al*, 2008). Based on these considerations, latex agglutination test (LAT) has been adopted as 1-day testing in the Florence screening programme (Castiglione *et al*, 2000), using a positivity threshold of 100 ng ml^−1^ Hb of sample solution; the same strategy has been then adopted by all Italian FOBT-based screening programmes (Zorzi *et al*, 2008).

Quantitative IFOBT assays (such as LAT), which allow the adjustment of positivity thresholds to fit screening aims are now available. Earlier studies (Itoh *et al*, 1996; Nakama *et al*, 1999, 2000; Castiglione *et al*, 2002; Edwards, 2005; Chen *et al*, 2007; Levi *et al*, 2007) have evaluated the impact of using different IFOBT positivity thresholds and single *vs* multiple sampling in terms of sensitivity/specificity ratio. The aim of this study is to provide further information on the diagnostic accuracy and colonoscopy workload of LAT-based screening using different positivity thresholds (⩾80, ⩾100 and ⩾120 ng ml^−1^), and single *vs* multiple samplings (1 *vs* 2 days).

## Materials and methods

This multicentric study involved four CRC population-based screening programmes of Northern (Alto Vicentino, Bussolengo and Feltre Local Health Units) and Central Italy (Florence ISPO Cancer Prevention and Research Institute), currently inviting residents aged 50–69 years to biennial IFOBT screening. The main characteristics of Italian screening programmes have already been described elsewhere (Zorzi *et al*, 2008). For this study, a special screening protocol was implemented. The study was submitted and approved by the Committees for Ethics of involved screening programmes. Participation to the study was offered to all subjects of target population who lived in those areas selected for the study. Subjects received a dedicated invitation letter, which invited them to screening and explained the aims of the study. An informed consent was required to all attending subjects who accepted to participate in the study. Attendees received two test tubes for collecting faeces from two consecutive bowel movements. People who refused to participate in the study could benefit from routine screening (one sample with positivity threshold at 100 ng ml^−1^). Tubes were marked to identify the first and the second samplings. Samples were collected and stored at 4°C until test development, usually within a week after collection.

Latex agglutination test assay (OC-Hemodia, Eiken, Tokyo, Japan, distributed by Alfa Wassermann, Milano, Italy) was employed. Quantitative assessment of human Hb was obtained through an antigen–antibody agglutination reaction using human anti-Hb polyclonal antibodies adsorbed on polystyrene particles: agglutination was measured as a 660-nm absorbency increase, proportional to Hb content in tested samples. The OC-Hemodia assay was developed by means of the OC-Sensor Micro instruments (Eiken, Tokyo, Japan), supplied by the distributor. Every screening programme had a reference laboratory where tests were processed by an own dedicated instrument. All the laboratories involved in the study cooperated in a national network for interlaboratory quality control, with the aim of monitoring the reproducibility of the FOBT technique.

Participants were instructed to return the test as soon as possible to avoid degradation of Hb in the samples. If the test could not be returned immediately, storage in a domestic refrigerator was advised.

Subjects with >79 ng ml^−1^ Hb, in at least one sample, were recalled for colonoscopy assessment, whereas subjects with <80 ng ml^−1^ Hb received a negative report by mail, recommending biennial rescreening. Incomplete colonoscopy prompted double contrast X-ray barium enema. Inadequate samples occurred in the absence of faecal material. No cutoff based on storage duration was used to define test as non-analysable. Subjects returning only one adequate sampling were excluded from the study. Subjects returning two inadequate samplings were advised to repeat the sampling.

The following screening strategies were compared: one sample with three different positivity thresholds (⩾80, ⩾100 and ⩾120 ng ml^−1^), and two samples with the same three thresholds, with at least one or both samples positive. One-day testing was determined as the result of the second sampling to simulate the actual exposure to degradation of Hb in a 1-day strategy.

Advanced adenoma was defined as any adenoma larger than 9 mm, and/or with a villous histological component higher than 20%, and/or with severe dysplasia. When more than one lesion was present, the subject was classified according to the most severe lesion. For this study, ‘significant neoplasia’ was defined, and is henceforth referred to as the sum of cancers and advanced adenomas. Performance was assessed according to positivity rate (PR=proportion of positive FOBT among subjects returning the samples), detection rate (DR=cancers or significant neoplasia detected among 1000 screened subjects) and positive predictive value (PPV=cancers or significant neoplasia detected among 100 FOBT+colonoscopy assessments). The number needed to scope (NTS) was calculated as the number of FOBT+colonoscopies needed to find one person with cancer or with significant neoplasia. Screening strategies were compared with the one presently adopted by Italian screening programmes (1-day testing at ⩾100 ng ml^−1^), henceforth referred to as ‘reference strategy’.

### Statistical analysis

Differences between tests were checked by *χ*^2^ method, significance being set at *P*<0.05. Differences between means were checked by the Student's *t*-test and analysis of variance (ANOVA). Confidences intervals at 95% (95% CI) of PR, DR and PPV were calculated using binomial distribution. To take into account multitesting, statistical comparisons among different strategies were carried out assuming the Bonferroni correction (Greenland and Rothman, 1998). Statistical analysis was performed using STATA 8.2 SE software.

## Results

From September 2005 to June 2007, 20 596 subjects aged 50–69 years in their first screening round were enrolled in the study. Twenty-two subjects returned only one FOBT sample were excluded from the study. Seventeen subjects returned two inadequate samplings were advised to repeat sampling. Out of them, 12 returned a double adequate sampling and they were included in the study. Two subjects returned again two inadequate samplings, whereas three subjects did not repeat any sampling. These latter five subjects were excluded from the study.

The principal characteristics of the study cohort are shown in Table 1. Mean attendance rate to the study was 56.2%, ranging from 49.1 to 77.9%. Mean acceptance rate to participate in the study for screening attendees was 96.1%.

Overall, the mean age was 59.9 years (s.d.=5.6), with no difference between programmes, apart from Florence (mean 61.0 years, s.d.=5.3), where a higher proportion of subjects aged 60–69 years were enrolled. Overall, a slight prevalence of women (53.8 *vs* 46.2%) was observed, even more so in the Florence programme (women=56.5%). Positivity rate varied among centres (range=6.7–9.2%) at a significant level (*P*<0.05). Average compliance to colonoscopy was 89.0%, ranging from 96.2% in Alto Vicentino to 83.5% in Florence. Average completed colonoscopy rate was 95.2%.

Positivity rate according to the different screening strategies is reported in Table 2. As expected, 1-day strategies with the lower (⩾80 ng ml^−1^) or higher (⩾120 ng ml^−1^) threshold were associated with a slight increase (+0.9%) or decrease (−0.5%) of PR, compared to the reference strategy, respectively. Positivity rates of 2-day strategies with at least one positive test were notably higher than the reference strategy, differences ranging from +1.4% (threshold ⩾120 ng ml^−1^) to +3.5% (threshold ⩾80 ng ml^−1^). Two-day strategies with both positive test results significantly decreased PR. All differences among each strategy and the reference strategy were statistically significant at *P*<0.00625 (threshold for significance after Bonferroni correction), except strategy of 1-day and cutoff ⩾120 ng ml^−1^ (*P*=0.01).

Overall, 69 cancers and 465 advanced adenomas were diagnosed. Cancer stage was I in 41 (59.4%), II in 13 (18.8%), III–IV in 15 (21.7%). No statistically significant difference in stage distribution was found among different strategies (*P*-values from 0.80 to 0.99), but the study was not designed and specifically not powered to find existing differences.

Detection rate for cancer and significant neoplasia are shown in Table 3. The reference strategy detected 2.8‰ cancers. Compared to the reference strategy, 2-day strategies with at least one positive result had a slightly higher DR for cancer (from +0.5 to +0.6‰, according to the positivity cutoff), whereas the more specific strategies (2-day with both positive results) had a slightly lower DR (from −0.5 to −0.6‰). No observed difference in DR reached statistical significance (*P=* 0.20–0.85).

The reference strategy detected 18.4‰ significant neoplasia, and more substantial differences in significant neoplasia DR were observed with respect to other strategies than for cancer DR. The most sensitive strategy (2-day with at least one positive sample at ⩾80 ng ml^−1^) allowed for an incremental DR of 7.5‰, whereas the most specific strategy (2-day with both positive samples at ⩾120 ng ml^−1^) decreased the DR by 6.3‰. After Bonferroni correction, all the observed differences in significant neoplasia DR among the reference strategy and strategies based either on both positive samples or at least one positive sample were statistically significant with the only exception of 1-day strategy at ⩾120 ng ml^−1^ (*P*=0.02).

Table 4A shows PPV for cancer and significant neoplasia with different strategies. The PPV of the reference strategy for cancer was 6.9%. All differences among each strategy and the reference strategy for cancer were not statistically significant at *P*<0.00625 (threshold for significance after Bonferroni correction) with *P*-values 0.00630–0.59.

The PPV of the reference strategy for significant neoplasia was 45.8%. After Bonferroni correction, all the observed differences in significant neoplasia PPV among the reference strategy and strategies based either on both positive samples or at least one positive sample were statistically significant with the only exception of 1-day strategy at ⩾120 ng ml^−1^ (*P*=0.0485).

As expected, lowering the threshold or adopting a 2-day strategy with at least one positive result reduced PPV; in particular, the introduction of a second sample was associated with a significant decrease of PPV for significant neoplasia (−9.4% in the 2-day strategy at ⩾80 ng ml^−1^). The most specific strategies, on the contrary, determined a statistically significant increase of PPV for significant neoplasia ranging from 12.6 to 20%, as the positivity threshold increased.

Table 4B shows NTS for cancer and significant neoplasia with different strategies. The NTS of the reference strategy for cancer and significant neoplasia was 14.5 and 2.2, respectively. The number needed to scope to find a significant neoplasia was lower than 2 for the 2-day strategies with both positive samples (1.7, 1.6 and 1.5 according to the threshold at 80, 100 or 120 ng ml^−1^, respectively). On the contrary, for the most sensitive 2-day strategies, the number of colonoscopy examinations needed to detect a significant neoplasia ranged from 2.4 to 2.7, by lowering the threshold from 120 to 80 ng ml^−1^. For the 1-day strategies, this indicator was about 2 (range=2.1–2.3).

Table 5 summarises the main results of the study according to the different strategies. The 2-day strategy with at least one positive result at ⩾100 ng ml^−1^ identified 21% more cancers and 26% more advanced adenomas than the reference strategy, whereas the 2-day strategy with at least one positive result at ⩾80 ng ml^−1^ allowed to diagnose a further amount of advanced adenomas (+18%). Increasing the threshold reduced the screening sensitivity, but at ⩾120 ng ml^−1^, it still allowed for a 17.5% cancer and 18% advanced adenoma incremental DR compared to the reference strategy. On the contrary, using a more specific 2-day strategy (with both samples at ⩾80, ⩾100 or ⩾120 ng ml^−1^) would miss 21, 30 and 34% significant neoplasia compared to the reference strategy, respectively.

In other words, compared to the reference strategy, the most sensitive strategy prompted 638 more colonoscopies and detected 12 more cancers and 143 more advanced adenomas. Therefore, the number needed to scope to detect one additional cancer was 53.2, whereas 4.5 colonoscopies were necessary to detect one additional advanced adenoma (see also Table 5). On the contrary, the most specific strategy prompted 447 less colonoscopies compared to the reference strategy, but missed 13 cancers and 116 advanced adenomas. Spared colonoscopies per each missed cancers were 34.4, those per each missed advanced adenomas were 3.9.

## Discussion

In recent years, a general agreement that IFOBT is superior to guaiac testing for screening purpose (Kahi *et al*, 2008; van Rossum *et al*, 2008) has grown. The IFOBT also allows for quantitative assay, and the positivity threshold may be adjusted to fit the local clinical and/or economic setting. This issue has been addressed in earlier studies (Nakama *et al*, 2000; Vilkin *et al*, 2005; Chen *et al*, 2007; Levi *et al*, 2007; Guittet and Launoy, 2008). In this study, we focused on the balance between cancer and advanced adenoma DR and colonoscopy workload by comparing different strategies using different number of samples and positivity thresholds, as assessed in a population-based screening service setting. To the best of our knowledge, this is the first study that simultaneously evaluated both these parameters in a large screening population sample.

Some slight differences in the distribution by age and sex were observed among centres in the study. A higher proportion of older (60–69 years) women in Florence compared to other centres is not due to difference in age distribution of the invited population, though a greater attendance in older women might be explained by the fact that in Florence programmes for cervical and breast cancer were activated many years ago and justifies higher awareness of older women usually less complying to screening invitations.

Differences occurred among the four centres as far as PR and DR are concerned. Such differences derived from the above-mentioned different composition for age and sex of examined population and probably from a different underlying incidence. Nevertheless, it does not affect the validity of the study. In fact, the aim of this study was to compare the performance of different FOBT strategies. If we consider the rate ratio (RR) between the most sensitive for significant neoplasia (subject invited to colonoscopy if at least one out of two sample was higher than 80 ng ml^−1^) and the less sensitive strategy (subject invited to colonoscopy if both samples resulted higher than 120 ng ml^−1^), we can observe that this ratio resulted quite stable among the four centres and without statistically significant differences. In fact, the RRs (on brackets the 95% CI) were 2.0 (1.5–2.5), 2.2 (1.7–2.7), 2.3 (1.7–3.3) and 2.3 (1.4–3.8) for Florence, Bussolengo, Alto Vicentino and Feltre, respectively.

It means that the results we can obtain for the overall study are substantially valid also within each centre.

From a public health point of view, the choice of the best screening test is a critical issue, which must take into account the existing healthcare organisation and local resources. So far, different choices have been adopted in countries where a CRC screening programme has been (or is going to be) implemented on a national basis. In Japan, the adopted strategy was biennial 2-day I-FOBT (Monoheam) at ⩾150 ng ml^−1^, based on the studies of Nakama *et al* ( 2000, 2001) suggesting that this strategy allows for the best cost/effectiveness ratio. In the UK pilot study (UK Colorectal Cancer Screening Pilot Group, 2004), a specific strategy was adopted with guaiac testing without rehydration. In Italy, a biennial strategy based on 1-day LAT at ⩾100 ng ml^−1^ has been adopted (Zorzi *et al*, 2008). This choice was based on several studies (Castiglione *et al*, 2000, 2002) and on an estimate of programme sensitivity for cancer, which confirmed a higher accuracy of 1-day IFOBT compared to guaiac testing (Zappa *et al*, 2001). On the whole, in respect to policies adopted in other European countries (UK Colorectal Cancer Screening Pilot Group, 2004), the Italian strategy (biennial one-time IFOBT at ⩾100 ng ml^−1^) favoured a sensitive approach, at the cost of reduced specificity.

Italian results have been confirmed by a recent study of van Rossum *et al*. (2008), which compared Hemoccult and OC-Hemodia in a randomised trial. Immunochemical faecal occult blood test showed a higher DR for cancer and advanced adenomas than guaiac test.

Comparing the performance of our reference strategy with the results of Dutch study, it can be noted that PR was substantially similar in the two settings, whereas our detection rates for cancer and advanced adenoma were lower than those found in the Dutch study (24 *vs* 18.4‰). Likewise, the PPV for significant neoplasia was higher in the van Rossum study compared to those registered in our study (51.8 *vs* 45.8%). However, comparison between the two studies should be taken with caution, as difference in detection rates could also relate to other confounding variables, such as a higher prevalence of lesions in Dutch population (Ferlay *et al*, 2007) compared to Italian population.

The reference strategy results in our study are consistent with those reported by the survey of Italian screening programmes (Zorzi *et al*, 2008) as far as diagnostic indicators (PR, DR and PPV) and compliance to assessment are concerned. This means that the population enrolled in this study is representative of the general population complying with the screening invitation, thus suggesting a safe generalisability of results.

One of the most important factors to be taken into account when choosing a screening strategy is the acceptability of the test. Several studies (Federici *et al*, 2005; van Rossum *et al*, 2008) showed a higher participation rate of invited subjects to 1-day IFOBT compared to 3-day guaiac screening. Several reasons may account for such a preference, such as the absence of dietary restrictions, a better hygiene in handling faecal samples and no need for multiple sampling.

In this study, no remarkable differences in participation rate were observed in every screening programmes involved in the study compared to that observed in resident subjects in the same areas, invited to the routine 1-day test screening.

Another important aspect of screening test acceptability is the easiness of sampling execution. Theoretically, multiple samplings should be more difficult to obtain and more unpleasant to handle, as subjects must store the first samples waiting the final one. In this study, only 22 of 20 596 recruited subjects did not return a second sample; this, together with the low rate of not acceptance to enter the study and the observation that attendance was similar to that registered by local service screening, using one-day sampling, suggests that the 2-day testing probably is not a major barrier to compliance with test returning.

It is well known that it takes many years for an advanced adenoma to progress (if at all) to CRC, and such a time window allows for cumulative sensitivity to be achieved by repeat screening. Therefore, a programme with a high compliance at repeat screening might favour a screening test with a lower one-shot sensitivity. On the contrary, when low compliance is expected at repeat screening, choosing a strategy with a high DR per single screening episode might be crucial.

A second relevant factor to be taken into account is the colonoscopy workload associated with a given strategy. Minimising PR is recommendable, as colonoscopy is an unpleasant and potentially harmful procedure, and accounts for a relevant part (about 50%) of total screening costs (Castiglione *et al*, 1997). The effect of different strategies on colonoscopy workload is quite variable; compared to the reference strategy, the number of required colonoscopies may vary from −54 (more specific) to +77% (more sensitive), whereas that of detected cancer may vary from −23 to +21%. Identifying the optimal strategy was not the aim of this study; however, although based on a limited sample size, our data suggest that minimising PR (by adopting the most specific strategy) is associated to a substantial loss in the DR, whereas, on the other hand, maximising the DR (by adopting the most sensitive strategy) is associated to hardly acceptable recall rate. Our study shows that different strategies modulate a continuum of opposite changes in sensitivity and specificity. Deciding the optimal cutoff, particularly in the absence of evidence of the efficacy of different strategies, is very difficult and may depend more on considerations of local resources (e.g., colonoscopy facilities) than purely on expected accuracy.

Colonoscopies needed to detect one cancer (i.e., the inverse of PPV) range from 21.2 with the less specific to 8.6 with the most specific strategy, whereas corresponding figures to detect one significant neoplasia (cancer+advanced adenoma) are between 2.7 and 1.5, respectively (see Table 5). In this study, recruited subjects were in their first screening examination. The PPV is expected to decrease in repeat screening, due to a lower prevalence of disease; such a decrease may not have an impact on the differences between different strategies, but a recent study by Van Rossum *et al* (2008) suggests to increase the positivity threshold when resources are limited and CRC prevalence is presumably low.

As far as sensitivity is concerned, it is worth noting that different strategies seem to have a greater impact on advanced adenomas rather than on cancer DR. In fact, the more sensitive strategy (2-day sampling, at least one sample ⩾80 ng ml^−1^) would obtain a 54% higher cancer DR (3.4 *vs* 2.1‰) compared to a less sensitive strategy (2-day sampling with both positive results at ⩾120 ng ml^−1^), whereas the DR for advanced adenomas would be more than double (+120%, 22.6 *vs* 10.0‰). The study of Guittet *et al* (2007) also supports the hypothesis that lowering IFOBT threshold will increase the DR of advanced adenomas more than the DR of cancer.

## Conclusions

In conclusion, none of the screening strategies analysed in our study showed a clear-cut superiority of results, but we observed a continuum of results for all performance indicators while passing from more specific to more sensitive strategies. The findings of this study do not provide an ultimate answer to which is the best CRC screening strategy, but they may provide an helpful guidance when deciding on the implementation screening protocols, and when considering how to adapt a screening strategy to the underlying context, based on CRC epidemiology, observed (or expected) programme performance with respect to compliance to invitation, DR and PPV and the available resources.

## Figures and Tables

**Table 1:** Main characteristics of subjects enrolled in the study by screening programme

**Programme**	**Florence**	**Bussolengo**	**Alto Vicentino**	**Feltre**	**Overall**
*Study population*					
Number of invited subjects	14 969	13 201	5768	2708	36 646
Participants to routine screening who refused to participate in the study (%)	3.6	4.5	4.1	4.0	3.9
Number of recruited subjects	7343	7211	3932	2110	20 596
Compliance to the study (%)	49.1	54.6	68.2	77.9	56.2
					
*Age class (%)*					
50–59 years	44.1	56.3	54.5	56.1	51.6
60–69 years	55.9	43.7	45.5	43.9	48.4
					
*Sex (%*)					
Male	43.5	47.4	48.1	48.0	46.2
Female	56.5	52.6	51.9	52.0	53.8
Subjects with a positive test (*n*)^a^	546	664	265	171	1646
Positivity rate (%)^b^	7.4	9.2	6.7	8.1	8.0
Compliance to colonoscopy (%)^c^	83.5	90.1	96.2	91.2	89.0
Colonoscopies performed (*n*)	456	598	255	156	1465
Complete colonoscopy rate (%)^d^	96.1	93.3	96.1	98.7	95.2
					
*Diagnosed lesions* (n)					
Cancers	24	26	12	7	69
Advanced adenomas	136	192	91	46	465

a At least one sample result at ⩾80 ng ml^−1^.

b Multiplied with 100 screened subjects.

c Multiplied with 100 positive subjects.

d Multiplied with 100 colonoscopies.

**Table 2:** Positivity rate (%) by screening strategy and difference with the reference strategy^a^

	**Positivity rate × 100 screened subjects**		
		**2–day strategy**	
**Cutoff**	**1-day strategy**	**(at least one sample with positive result)**	**(both samples with positive results)**
⩾80 ng ml^−1^	5.5	8.0	2.8
	+0.9 (0.5–1.4)	+3.5 (3.0–3.9)	−1.7 (−2.1 to −1.4)
⩾100 ng ml^−1^	4.5^a^	6.7	2.3
	(reference)	+2.2 (1.7–2.6)	−2.2 (−2.6 to −1.9)
⩾120 ng ml^−1^	4.0^b^	5.9	2.0
	−0.5 (−0.9 to −0.1)	+1.4 (1.0–1.8)	−2.5 (−2.8 to −2.2)

Statistical comparison of strategies (with Bonferroni correction for multitesting).

95% CI (confidence interval) of difference is given in brackets.

All differences among each strategy and the reference strategy were statistically significant at *P*<0.00625 (threshold for significance after Bonferroni correction), except strategy of 1-day and cutoff ⩾120 ng ml^−1^.

a Reference strategy: 1-day and cutoff ⩾100 ng ml^−1^.

b *P*=0.0113.

**Table 3:** Detection rate (‰) for cancer and for significant neoplasia^a^ by screening strategy and difference with the reference strategy^b^

	**Detection rate × 1000 screened subjects**		
		**2–day strategy**	
**Cutoff**	**1-day strategy**	**(at least one sample with positive result)**	**(both samples with positive results)**
*Cancer*			
⩾80 ng ml^−1^	2.9	3.4	2.3
	+0.1 (−0.9 to 1.1)	+0.6 (−0.5 to 1.7)	−0.5 (−1.5 to 0.5)
⩾100 ng ml^−1^	2.8^b^	3.4	2.1
	(reference)	+0.6 (−0.5 to 1.7)	−0.6 (−1.6 to 0.3)
⩾120 ng ml^−1^	2.7	3.3	2.1
	−0.1 (−1.1 to 1.0)	+0.5 (−0.6 to 1.5)	−0.6 (−1.6 to 0.3)
			
*Significant neoplasia*			
⩾80 ng ml^−1^	20.7	25.9^c^	14.6^c^
	+2.3 (−0.4 to 5.0)	+7.5 (4.7–10.4)	−3.8 (−6.3 to −1.3)
⩾100 ng ml^−1^	18.4^b^	23.1^c^	12.8^c^
	(reference)	+4.7 (1.9–7.4)	−5.6 (−8.0 to −3.2)
⩾120 ng ml^−1^	17.3	21.7^d^	12.1^c^
	−1.1 (−3.6 to 1.5)	+3.3 (0.6–6.0)	−6.3 (−8.6 to −3.9)

Statistical comparison of strategies (with Bonferroni correction for multitesting).

95% CI (confidence interval) of difference is given in brackets.

a Significant neoplasia: cancer+advanced adenoma (any adenoma larger than 9 mm, and/or with a villous histological component higher than 20%, and/or with severe dysplasia).

b Reference strategy: 1-day and cutoff ⩾100 ng ml^−1^.

c Differences among strategy and the reference strategy were statistically significant at *P*<0.00625 (threshold for significance after Bonferroni correction).

d Non-significant at *P*<0.00625 (*P*=0.0168).

**Table 4:** Positive predictive value (PPV) (%) of FOBT+ colonoscopy for cancer and for significant neoplasia^a^ and number needed to scope (NTS) to find a cancer or a significant neoplasia by screening strategy and difference with the reference strategy^b^

		**2–day strategy**	
**Cutoff**	**1-day strategy**	**(at least one sample with positive result)**	**(both samples with positive results)**
(A) PPV × 100 colonoscopies			
*Cancer*			
⩾80 ng ml^−1^	5.9	4.7^**^	9.1
	−1.0 (−3.3 to 1.3)	−2.2 (−4.2 to −0.1)	+2.2 (−0.8 to 5.3)
⩾100 ng ml^−1^	6.9^b^	5.7	10.4^***^
	(reference)	−1.2 (−3.4 to 0.9)	+3.5 (0.1 to 6.9)
⩾120 ng ml^−1^	7.6	6.2	11.6^****^
	+0.7 (−1.9 to 3.3)	−0.7 (−3.0 to 1.6)	+4.7 (1.0 to 8.3)
			
*Significant neoplasia*			
⩾80 ng ml^−1^	42.7	36.5	58.5
	−3.2 (−7.8 to 1.4)	−9.4 (−13.6 to −5.2)^c^	+12.6 (7.2–18.1)^c^
⩾100 ng ml^−1^	45.8	38.9	62.4
	(reference)	−6.9 (−11.3 to −2.6)^c^	+16.6 (10.9–22.3)^c^
⩾120 ng ml^−1^	48.4	41.3^*****^	65.8
	+2.6 (−2.3 to 7.6)	−4.5 (−9.0 to 0.0)	+20.0 (14.1–25.8)^c^
			
(B) NTS^d^			
*Cancer*			
⩾80 ng ml^−1^	17.0 (13.6–22.6)	21.2 (17.3–27.6)	11.0 (8.6–15.1)
⩾100 ng ml^−1^	14.5 (11.6–19.4)^b^	17.7 (14.4–23.0)	9.6 (7.5–13.4)
	(reference)		
⩾120 ng ml^−1^	13.2 (10.5–17.6)	16.2 (13.1–21.0)	8.6 (6.8–12.0)
			
*Significant neoplasia*			
⩾80 ng ml^−1^	2.3 (2.2–2.5)	2.7 (2.6–2.9)	1.7 (1.6–1.9)
⩾100 ng ml^−1^	2.2 (2.0–2.4)^b^	2.6 (2.4–2.8)	1.6 (1.5–1.7)
	(reference)		
⩾120 ng ml^−1^	2.1 (1.9–2.2)	2.4 (2.3–2.6)	1.5 (1.4–1.6)

Statistical comparison of strategies (adopting Bonferroni correction for multitesting). 95% CI (confidence interval) of difference is given in brackets.

a Significant neoplasia: cancer+advanced adenoma (any adenoma larger than 9 mm, and/or with a villous histological component higher than 20%, and/or with severe dysplasia).

b Reference strategy: 1-day and cutoff ⩾100 ng ml^−1^.

c Differences among strategy and the reference were statistically significant at *P*<0.00625 (threshold for significance after Bonferroni correction).

Non-significant at *P*<0.00625, ^*^P=0.0277, ^**^P=0.0312, ^***^P=0.0063, ^****^P=0.0485.

d NTS: number of FOBT+colonoscopies needed to find a cancer or a significant neoplasia.

**Table 5:** Positivity rate (PR), number of screen detected cancers and advanced adenomas, number of colonoscopies, detection rate (DR) of cancers and advanced adenomas (per 1000 screened subjects) and number needed to scope (NTS)^a^ to find a cancer or a significant neoplasia^b^ by screening strategy

**Strategy**		**PR%**	**Number (difference %)**			**DR (‰)**		**NTS^a^**	
**Positive samples**	**Cutoff (ng ml^−1^)**		**Cancer**	**Advanced adenomas**	**Colonoscopies**	**Cancer**	**Advanced adenomas**	**Cancer**	**Significant neoplasia^b^**
At least one	⩾80	8.0	69 (+21.1)	465 (+44.4)	1465 (+77.2)	3.4	22.6	21.2	2.7
At least one	⩾100	6.7	69 (+21.1)	406 (+26.1)	1221 (+47.6)	3.4	19.7	17.7	2.6
At least one	⩾120	5.9	67 (+17.5)	380 (+18.0)	1082 (+30.8)	3.3	18.5	16.2	2.4
One	⩾80	5.5	59 (+3.5)	368 (+14.3)	1001 (+21.0)	2.9	17.9	17.0	2.3
One^c^ (reference)	⩾100^c^	4.5	57	322	827	2.8	15.6	14.5	2.2
One	⩾120	4.0	56 (−1.8)	301 (−6.5)	737 (−10.9)	2.7	14.6	13.2	2.1
Both	⩾80	2.8	47 (−17.5)	254 (−21.1)	515 (−37.7)	2.3	12.3	11.0	1.7
Both	⩾100	2.3	44 (−22.8)	220 (−31.7)	423 (−48.9)	2.1	10.7	9.6	1.6
Both	⩾120	2.0	44 (−22.8)	206 (−36.0)	380 (−54.1)	2.1	10.0	8.6	1.5

Differences (%) of number of cancers, advanced adenomas and colonoscopies with reference strategy are given in brackets.

a NTS: number of FOBT+ colonoscopies needed to find a cancer or a significant neoplasia.

b Significant neoplasia: cancer+advanced adenomas (any adenoma larger than 9 mm, and/or with a villous histological component higher than 20%, and/or with severe dysplasia).

c Reference strategy: 1-day and cutoff ⩾100 ng ml^−1^.
